# Benefits and barriers of home blood pressure monitoring in pregnancy: perspectives of obstetric doctors from a Ghanaian tertiary hospital

**DOI:** 10.1186/s12884-023-05363-5

**Published:** 2023-01-19

**Authors:** Namratha Atluri, Titus K. Beyuo, Samuel A. Oppong, Sarah D. Compton, Cheryl A. Moyer, Emma R. Lawrence

**Affiliations:** 1 University of Michigan Medical School, 1301 Catherine St, MI 48109 Ann Arbor, USA; 2 Department of Obstetrics and Gynaecology, University of Ghana Medical School, Korle Bu, Accra, P.O. Box 4236, Ghana; 3 Department of Obstetrics and Gynecology, University of Michigan, 1500 E. Medical Center Dr, 48109 Ann Arbor, MI USA

**Keywords:** Preeclampsia, Eclampsia, LMIC, Sub-Saharan Africa, Home monitoring, Patient monitoring, Provider perspective, High blood pressure in pregnancy, Hypertensive disorders of pregnancy

## Abstract

**Background:**

Delayed diagnosis of preeclampsia contributes to maternal morbidity and mortality. Patient-performed home blood pressure monitoring facilitates more frequent monitoring and earlier diagnosis. However, challenges may exist to implementation in low- and middle income-countries.

**Methods:**

This cross-sectional mixed methods study evaluated obstetric doctors’ perspectives on the benefits of and barriers to the implementation of home blood pressure monitoring among pregnant women in Ghana. Participants were doctors providing obstetric care at Korle Bu Teaching Hospital. Electronic surveys were completed by 75 participants (response rate 49.3%), consisting of demographics and questions on attitudes and perceived benefits and challenges of home BP monitoring. Semi-structured interviews were completed by 22 participants to expand on their perspectives.

**Results:**

Quantitative and qualitative results converged to highlight that the current state of blood pressure monitoring among pregnant women in Ghana is inadequate. The majority agreed that delayed diagnosis of preeclampsia leads to poor health outcomes in their patients (90.6%, *n* = 68) and earlier detection would improve outcomes (98.7%, *n* = 74). Key qualitative benefits to the adoption of home blood pressure monitoring were patient empowerment and trust of diagnosis, more quantity and quality of blood pressure data, and improvement in systems-level efficiency. The most significant barriers were the cost of monitors, lack of a communication system to convey abnormal values, and low health literacy. Overall, doctors felt that most barriers could be overcome with patient education and counseling, and that benefits far outweighed barriers. The majority of doctors (81.3%, *n* = 61), would use home BP data to inform their clinical decisions and 89% (*n* = 67) would take immediate action based on elevated home BP values. 91% (*n* = 68) would recommend home BP monitoring to their pregnant patients.

**Conclusion:**

Obstetric doctors in Ghana strongly support the implementation of home blood pressure monitoring, would use values to inform their clinical management, and believe it would improve patient outcomes. Addressing the most significant barriers, including cost of blood pressure monitors, lack of a communication system to convey abnormal values, and need for patient education, is essential for successful implementation.

**Supplementary Information:**

The online version contains supplementary material available at 10.1186/s12884-023-05363-5.

## Introduction

Hypertensive disorders of pregnancy (HDP) are serious complications of pregnancy that contribute to poor maternal and neonatal outcomes [1, 2]. HDP exist along a spectrum spanning from gestational hypertension (blood pressures > 140/90 diagnosed after 20 weeks gestation), preeclampsia without severe features (gestational hypertension plus proteinuria), preeclampsia with severe features (preeclampsia plus symptoms, blood pressures > 160/ 100, or laboratory derangements), and eclampsia (preeclampsia with severe features plus seizures). Most HDP-related deaths can be prevented by early detection and timely medical interventions [2–4]. However, many barriers to adequate obstetric care exist in low-middle-income countries (LMICs), and strikingly, 99% of maternal deaths from HDP occur in LMICs [5–8]. Poor antenatal clinic attendance, long intervals between routine visits, and the low rates of preeclampsia diagnosed prior to the onset of eclamptic seizures suggest a need for improved models of blood pressure (BP) monitoring in such low-resource settings [2, 4, 9–12].

Home BP monitoring, which involves patients measuring their own BPs outside of a clinical setting, has been successfully implemented in many high-income countries (HICs) with demonstrated patient accuracy and adherence [13–15]. Existing evidence suggests that benefits to home BP monitoring include reduced healthcare resource use and high patient and clinician satisfaction [14–17]. Studies in non-pregnant patients with chronic hypertension demonstrate improvement in blood pressure control with home blood pressure monitoring [18, 19]. While home BP monitoring in pregnancy has not yet been proven to improve clinical outcomes [20, 21], it still may be of particular benefit in LMIC settings that have a high prevalence of HDP and barriers to providing regular, in-person care. However, challenges to implementation may exist.

This study aims to understand the feasibility of home BP monitoring among pregnant patients in urban Ghana, as perceived by obstetric doctors. Since doctors can identify challenges from multiple levels (patient, clinical, and system-level) and because doctor buy-in is essential to the successful implementation of any clinical intervention, this study intended to evaluate doctor perspectives on benefits and barriers to home BP monitoring among pregnant women in Ghana.

## Materials and methods

### Setting

This mixed methods study was conducted at the Korle Bu Teaching Hospital (KBTH), Ghana’s largest tertiary care hospital. The Department of Obstetrics and Gynaecology (OBGYN) at KBTH runs a six-floor maternity unit with 275 inpatient beds, and manages approximately 10,000 deliveries annually. 15% of deliveries are complicated by HDP, which is the leading cause of maternal mortality at KBTH [22].

### Participants

Participants were doctors who provide obstetric care, including antenatal care, intrapartum care, and postpartum care, at KBTH. Inclusion criteria were doctors who provide care to obstetric patients, whose primary site of clinical work is KBTH, and who have experience diagnosing and managing patients with HDP. There were no exclusion criteria. At KBTH, obstetric care is provided by three groups of doctors: (1) house officers (doctors who recently graduated from medical school and are rotating through core clinical services including OBGYN), (2) OBGYN residents (doctors who have completed their house officers training and are now undergoing specialty training in OBGYN), and (3) OBGYN consultants (doctors who have completed specialty training in OBGYN) known in other settings as “attendings”.

### Recruitment

Survey participants were identified using KBTH’s OBGYN departmental roster which lists all doctors currently providing obstetric care at KBTH. The survey was administered electronically by posting the survey link to three WhatsApp groups at KBTH: (1) group for house officers rotating on OBGYN (2) group for OBGYN residents (3) group for OBGYN attendings. At KBTH, WhatsApp groups are used for workplace communication, coordination of departmental events, and research. In total, there were 152 members in these groups, which was consistent with the department rosters. Seventy-five surveys were completed with a response rate of 49.3% (Table 1).

Interview participants were identified using KBTH’s OBGYN departmental roster and the guidance of a local research team member. Purposive sampling was employed to identify obstetric providers with meaningful experience managing patients with HDP and ensure diversity of clinical roles. For each interview, transcription and review was conducted in an ongoing manner, and the final number of participants was determined by thematic saturation of data. A total of 22 participants (4 house officers, 6 junior residents, 8 senior residents, and 4 consultants) were interviewed (Table 1).

**Table 1:** Demographics of survey and interview participants

**Characteristic**	**Survey participants (*n* = 75)** Frequency (proportion)	**Interview participants (*n* = 22)** Frequency (proportion)
Clinical role		
House Officer	23 (30.7%)	4 (18.2%)
Junior Resident in	17 (22.7%)	6 (27.3%)
Obstetrics/Gynaecology	25 (33.3%)	8 (36.4%)
Senior Resident in	10 (13.3%)	4 (18.2%)
Obstetrics/Gynaecology		
Consultant (“attending”) in Obstetrics/Gynaecology		
Gender		
Male	54 (73.0%)	18 (81.8%)
Female	20 (27.0%)	4 (18.2%)
Other/ Prefer Not To Respond	0 (0%)	0 (0%)
Years in practice as a doctor		
< 1	21 (28.0%)	4 (18.2%)
1—5	5 (6.7%)	2 (9.1%)
6—10	19 (25.3%)	8 (36.4%)
11—20	25 (33.3%)	7 (31.8%)
> 20	5 (6.7%)	1 (4.5%)
Average patients with preeclampsia managed **weekly**		
0—5	20 (27.0%)	5 (22.7.0%)
6—10	40 (54.1%)	8 (36.4%)
11—15	8 (10.8%)	4 (18.2%)
16—20	3 (4.1%)	5 (22.7%)
> 20	3 (4.1%)	0 (0.0%)
Average patients with eclampsia managed **monthly**		
0	7 (9.3%)	3 (13.6%)
1	28 (37.3%)	7 (31.8%)
2	12 (16.0%)	7 (31.8%)
3	11 (14.7%)	5 (22.7%)
4	4 (5.3%)	0 (0.0%)
5 or higher	13 (17.3%)	0 (0.0%)

### Design

Our mixed methods study consisted of surveys and semi-structured interviews. Given the absence of validated scales to evaluate barriers and benefits of home BP monitoring, our survey questions were newly developed for this research. Survey questions were based on the local expertise of our authors as practicing OBGYNs, and were piloted for clarity and content prior to finalization and administration. Our semi-structured interview guide was anchored in grounded theory, a qualitative approach for collecting and analyzing data without imposing previously constructed theoretical frameworks. This approach was used to capture participants’ perspectives without assuming they would conform to the researchers’ ideas about home blood pressure monitoring. Qualitative trustworthiness was established based on the credibility of the participants as obstetric providers.

### Procedures

An electronic survey was developed in RedCAP, an online program for secure survey development and data management. The survey link was electronically distributed to WhatsApp groups as detailed earlier and survey responses were entered by participants electronically. Survey questions were in English, the language used for medical education in Ghana. Surveys were organized into 4 sections and consisted of closed-ended questions with categorical response options (Supplemental file 1). The first section asked demographic questions about the participant, their clinical role, and their clinical experience with preeclampsia and eclampsia. The second section focused on attitudes about the impact and management of preeclampsia, consisting of 5 statements with Likert scale responses (strongly agree to strongly disagree). The third section asked about the implementation of home BP monitoring, including anticipated patient ability and accuracy, provider attitudes toward trust and use of home BP values and perspectives on feasibility and overall recommendation of use. This section included 14 statements with Likert scale responses (strongly agree to strongly disagree). The final section focused on anticipated barriers to home BP monitoring and asked participants to indicate the relative importance of six barriers on a scale from 1 (minimum barrier) to 5 (maximal barrier). This list of barriers was determined by the authors based on their pre-study hypotheses and informed by local experience in Ghana.

A semi-structured interview tool asked about providers’ experiences, attitudes, and perceived benefits and challenges to implementing home blood pressure monitoring among their pregnant patients in Ghana (Supplemental file 2). Interviews were conducted by an American female medical student who received training on qualitative interviewing techniques and cultural humility. Interviews were conducted in English, face-to-face, from January 27th to February 17th, 2022 in various quiet spaces within the KBTH compound. They lasted approximately 20–40 min. Ethical approval was granted from KBTH (KBTH-STC 00098/2021) and University of Michigan (HUM00200589). Written informed consent was obtained from all participants. No incentive was offered for participation.

### Analysis

A convergent mixed-methods design was used for analysis; quantitative and qualitative data were first analyzed separately and then merged for comparison [23]. Stata (Version 16.0 StataCorp. 2019) was used for analysis of survey data. Demographic data was summarized using frequencies and proportions. Descriptive analysis was conducted for survey responses, consisting of frequencies and proportions for Likert scale responses and mean and standard deviation for the barriers scale responses. Interviews were audio-recorded and transcribed verbatim in English. Transcripts were uploaded into NVivo 12.0 for qualitative coding. Transcripts were independently reviewed by two researchers who each developed a preliminary set of codes. Then, the two researchers employed an incremental and iterative process to review the transcripts together and collectively develop a codebook consisting of stabilized keyword-phrases. The transcripts were then coded using the codebook. Finally, the coded transcripts were thematically analyzed using the Attride-Sterling Framework for qualitative analysis, consisting of a framework of basic, organizing, and global themes [24].

## Results

**Fig. 1:**
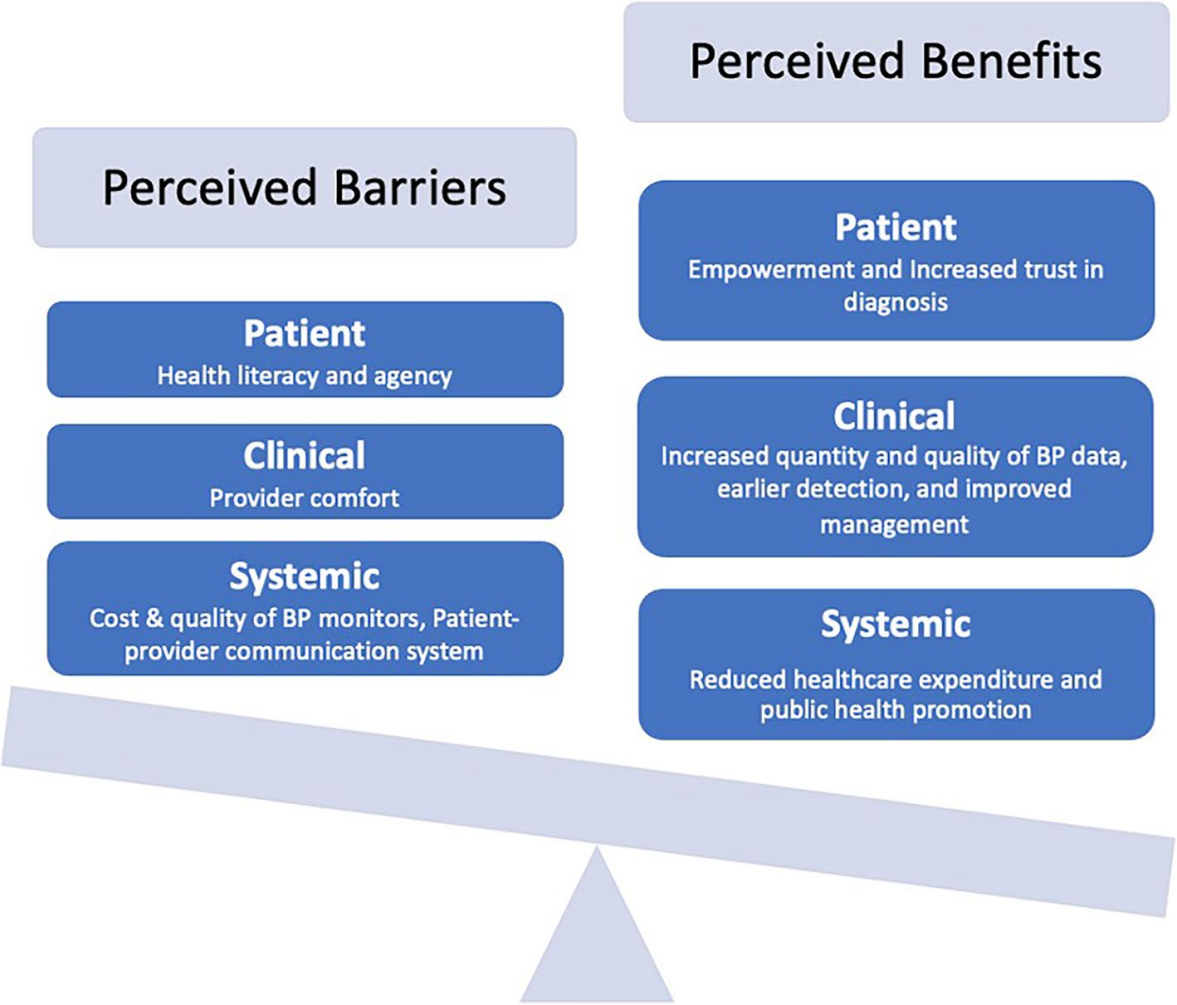
Benefits of Home Blood Pressure Monitoring Outweigh the Barriers

Quantitative and qualitative results converged to highlight that the current state of BP monitoring among pregnant women in Ghana is inadequate. Doctors perceived several patient-level, clinical-level, and systems-level benefits to the adoption of home BP monitoring but also emphasized key challenges to implementation that must be recognized and addressed. Overall, doctors felt that most barriers could be overcome with patient education and counseling and that benefits far outweighed barriers (Fig. 1).

### Current state of BP monitoring among pregnant women in Ghana is inadequate

In surveys, the vast majority of doctors agreed that maternal morbidity and mortality from preeclampsia is preventable (98.7%, *n* = 74), delayed diagnosis of preeclampsia leads to poor health outcomes amongst their patients (90.6%, *n* = 68), and early detection of preeclampsia would reduce poor health outcomes (98.7%, *n* = 74). In interviews, many doctors identified issues with the current quality and frequency of BP monitoring performed among pregnant women that inevitably leads to delayed detection and poor outcomes. In-hospital BP measurements are of poor quality due to a variety of reasons, including the use of ill-calibrated/ unvalidated machines, inappropriate cuff size (usually only one standard cuff size is available for all patients), and improper technique. Inadequate frequency of BP monitoring is also a problem, with long intervals between scheduled antenatal clinic visits, especially when compounded by poor antenatal clinic attendance by patients at baseline.

*“So it means that when you come [for antenatal care], the next time you’re coming is a month later. So, within that time, what are you doing? Who is monitoring your BP and all that? So if anything happens within that period, you don’t know. Or no one will know.” ID 13, Male Junior Resident*

Survey data suggested that while 63% (*n* = 46) of participants were aware that high-risk pregnant patients in many countries monitor their BPs at home, only 21.7% (*n* = 16) have experienced their own patients in Ghana engaging in home BP monitoring. Most pregnant women in Ghana do not have BP monitors at home and are not aware they could have elevated BPs if they have no symptoms.

*“Most people don’t have BP machines at home. And they just live life on a day-to-day basis. Unless you have symptoms, you don’t really go to the hospital. You don’t see the need to check your blood pressure.” ID 16, Female House Officer*

Despite the low utilization of home BP monitoring, several doctors have still had positive experiences with the small number of patients who do monitor their BPs outside of the hospital, either by using their own monitor at home or by visiting a nearby pharmacy daily. They noted many advantages, including cost and convenience, to patients using their own monitor at home versus visiting a pharmacy.

*“So they may have to walk to a pharmacy shop and get their blood pressures checked. But then, if they have to do this several times a day, it becomes a bit of an inconvenience. And of course, it has financial implications because they have to pay for their blood pressures to be monitored. So it’s easier if they have the education and they have the means to get a blood pressure monitor. Then they can do it themselves.” ID 1, Male Senior Resident*

### Doctors perceived significant benefits to home BP monitoring

Doctors anticipated many benefits to wider-scale implementation of home BP monitoring (Table 2), with clinical benefits perceived as the most important.

**Table 2:** Anticipated benefits of home BP monitoring

Benefit categories	Benefits	Representative quotation
**Patient Benefits**	**Empowerment**	“So she owns her health. She understands, she asks questions. It's more of a joint care, multidisciplinary approach. We have the patient as one of the clinicians.” ID 17, Female Senior Resident
	**Trust of diagnosis**	“When [patients] start home BP monitoring early, they realize that it's not the doctor saying it…you know this is something I didn't have and I can see myself that [BP] is going up…As against me springing a diagnosis on you.” ID 4, Female Consultant
**Clinical Benefits**	**More quantity and quality of data**	“I think [home monitoring] would create a better overall picture of the patient's response rather than that snapshot that you get when they come to you once every 4 weeks.” ID 22, Male Consultant
	**Earlier detection and improved management**	“If the high risk people are monitoring at home,…we are going to pick most of the diagnoses early, it will help us put in interventions early, and we are definitely going to achieve better outcomes.” ID 12, Male Junior Resident
**Systemic Benefits**	**Reduced healthcare expenditure**	"So [home monitoring] will probably help reduce the costs for the facility and for the patient. It will help us catch late cases earlier. It will reduce the time and then probably the manpower you put into managing those adverse cases.” ID 13, Male Junior Resident
	**Public health promotion**	“Other members of the family are going to go ahead and check their blood pressures. And they could have essential hypertension that could be picked up and they could seek care. So giving it to one person will save the whole family.” ID 10, Male House Officer

In interviews, doctors unanimously described how the ability to gain a greater quantity and quality of BP data would likely lead to improved health outcomes. Doctors noted that the greater quantity of readings obtained with home BP monitoring, as opposed to a singular value obtained at in-person ANC visits, would enable them to see trends throughout pregnancy and adjust medications as necessary. Since patients are comfortable in their home environment without the stresses of a busy hospital, home BP monitoring was also thought to be less affected by “white coat hypertension” (the idea that being around providers can make patients anxious and thus elevate their BP) and more representative of a patient’s true clinical status. The notion of improved health outcomes centered on the widely held belief that home BP monitoring would allow for earlier detection of newly elevated BPs. While the overall detection of preeclampsia may increase, potentially leading to more patients at the hospital to care for, doctors viewed this positively. They expected home BP monitoring to lead to a reduction in the progression to eclampsia and incidence of complications, enabling them to provide better care to fewer acutely ill patients.

Other benefits often described by doctors included patient-level benefits of empowerment, increased involvement in their own healthcare, and improved acceptance of their diagnosis when new elevated BPs are detected. System-level benefits included reduced healthcare resource expenditure due to earlier diagnosis and prevention of complications and collateral public health benefits to family members who may also start checking their BPs (Table 2).

### Doctors acknowledged key barriers to home BP monitoring

**Fig. 2:**
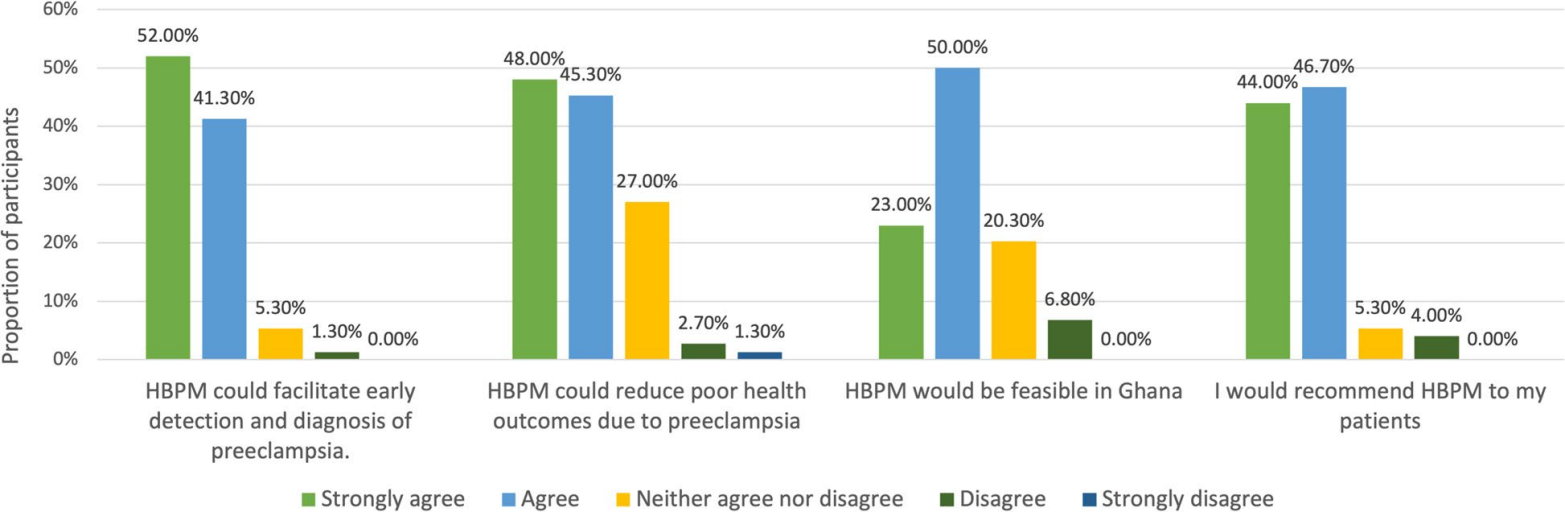
Magnitude of Barriers to Implementation of Home Blood Pressure Monitoring

The most important systems-level barrier, and the single most significant barrier overall, was the ability of patients to afford and access home BP monitors (Table 3; Fig. 2).

**Table 3:** Potential barriers to home BP monitoring

Barrier categories	Barriers	Representative qualitative quotation
**Patient Barriers**	**Health literacy**	“I think the number one barrier is when they've not been properly educated. If they don't understand. Once they have the understanding, I believe they will use it. So the next thing will be working on their understanding. So lots of counseling.” ID 21, Male Consultant
	**Agency to make and act upon healthcare decisions**	“Here is a case that a lady comes, she's told about everything. But the husband doesn't come. The husband doesn't see the need to come and listen to what we say. Meanwhile, he is the provider. Yes, so if you say, okay, let's get a [BP monitor],….[he] tells you that I don't have the money to buy the [BP monitor] or I won't do it. He's taking the major decision here.” ID 9, Male Senior Resident
	**Willingness to perform regular BP monitoring**	“Every woman wants to have a safe delivery and to have their babies. So, if they understand the complications of the disease, they will be motivated.” ID 11, Male Senior Resident
	**Ability to use BP monitor**	“If they have been taught well…I think our patients are smart enough to do it and it should be accurate.” ID 6, Male Senior Resident
**Clinical Barriers**	**Provider comfort with home management**	“If something goes wrong, we will be held responsible. Because you allowed her to go home and now she has had a stroke. So I think from healthcare workers, that may be the resistance to allowing more women to monitor their blood pressures at home. If something goes wrong, who is going to be held responsible?” ID 4, Female Consultant
	**Provider trust in home BP values**	“Most patients who understand what is wrong with them, will not lie…I think that is the bottomline. By and large I think most people will not give false readings.” ID 14, Male Junior Resident
**Systemic Barriers**	**Cost of BP monitors**	“When it comes to our care, cost is literally a big barrier. You know most patients cannot afford [a BP monitor].” ID 19, Male Senior Resident
	**Quality of BP monitors**	“You have to check to see the cuff size, whether it tallies. Whether the machine is working well or not. Use it on another person to be really sure that hey this machine is good. It's not just that the values are high, the machine is actually appropriate for the person.” ID 5, Male Junior Resident
	**Provider-patient communication of abnormal BP values**	“I think [there should be] systems in place so that when BPs are elevated…that the [patient] can call and complain to somebody that this is the BP I checked and these are the symptoms I am feeling and all of that. And if they need for the person to [come to the hospital], then the person reports.” ID 8, Male Junior Resident

Doctors felt most patients would be unable to purchase a monitor unless it were well-subsidized or free. Additionally, doctors worried about the quality of monitors that patients may buy with regards to calibration, validation, and appropriate cuff sizes. They also expressed concerns about future maintenance and battery life. However, doctors felt that the cost of providing home BP monitors to patients is well justified, and it would be less expensive to provide a BP monitor for home use than admit a patient to the hospital simply for blood pressure monitoring.

*“It’s the whole cost and benefit analysis. If you can provide pregnant women with a device that could prevent her from coming to the hospital and be admitted, and then the cost of reporting to the hospital, the admission, daily bed charges...in the long run you realize it’s actually cheaper to actually provide a device.” ID 17, Female Senior Resident*

The second main systems-level barrier doctors highlighted was the lack of an efficient system for patient-provider communication (Table 3; Fig. 2). Currently, a central triage phone system does not exist for patients to communicate abnormal home BP values or other concerns. Instead, they either call their doctor’s personal phone number or come into the hospital for assessment. Doctors identify the limitations in this current approach, which depends on the 24 − 7 availability of individual physicians, or long transport times to a healthcare facility. Doctors viewed not having a protocol with clear next steps for patients when they detect home BP elevations as a potential challenge to the utility of home BP monitoring. However, doctors also believed that with proper planning and training of staff, such a protocol and communication system can be developed.

While systemic barriers were viewed as most important, doctors did report several patient-level barriers (Table 3; Fig. 2). Interviewed doctors reported concerns about patients’ sufficient health literacy to recognize elevated BP values and agency to take action when elevated values are detected. Consistent with interviews, surveys showed that only half of participants felt patients know about the risks and complications of preeclampsia (44%, *n* = 33), could follow the recommended schedule for monitoring BPs at home (61%, *n* = 45), and could accurately monitor BPs at home (48%, *n* = 36). Doctors expanded that patients who may not truly appreciate the risks of preeclampsia may not adhere to home BP monitoring as recommended (not regularly measure blood pressures or report dishonest values). Importantly, all doctors during interviews who mentioned these potential patient-level challenges also stated that they can be overcome with proper counseling, focused education by ancillary health staff during antenatal visits, and additional public health initiatives. Notably, interviewed doctors did not view patient willingness to use BP monitors to be significant barriers for the majority of their patients, with surveys showing 77% (*n* = 58) of participants believing patients would be interested in monitoring BPs at home.

Clinical barriers were overall less important and included provider discomfort managing patients at home due to concern for poor outcomes occurring at home and subsequent blame from colleagues, as well as potential distrust of patient-measured home blood pressure values (Table 3). Similar to patient-level barriers, doctors felt that these could be overcome with patient education and counseling.

### Doctors believed home BP monitoring would be feasible and impactful

**Fig. 3:**
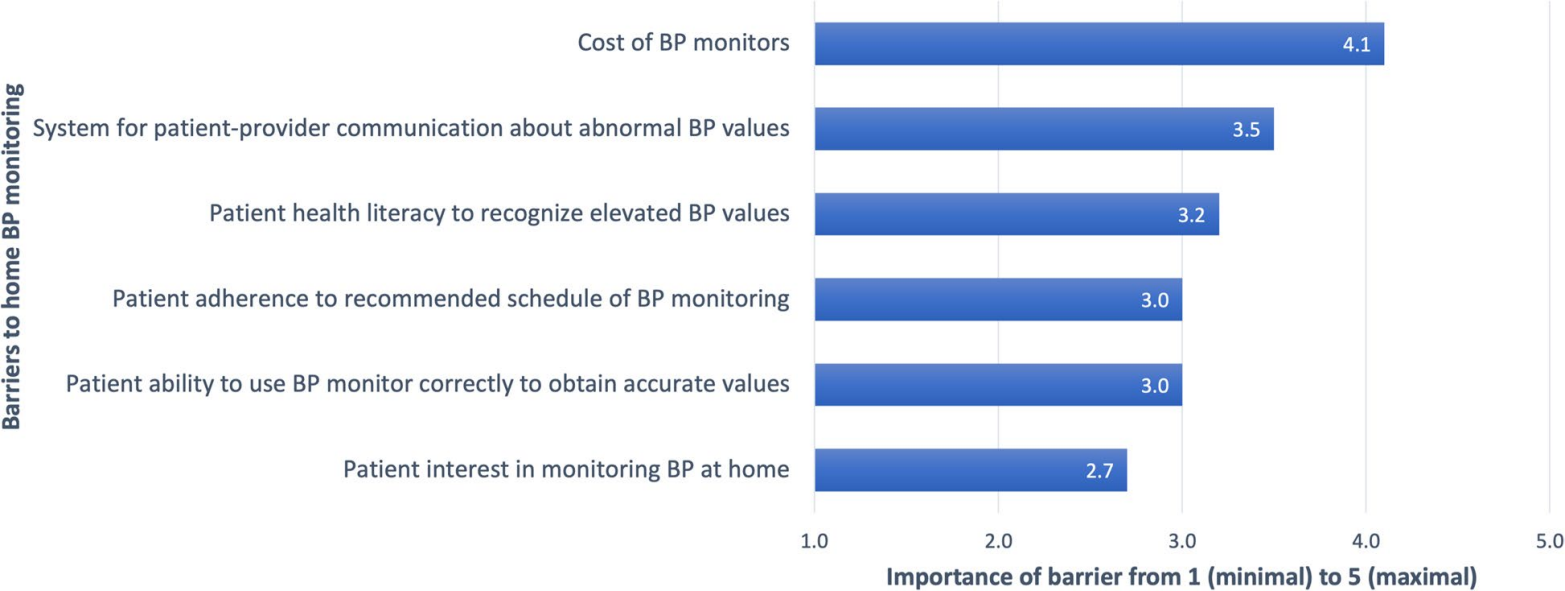
Physician Views on Utility of Home Blood Pressure Monitoring

After considering the benefits and barriers, most doctors in interviews and surveys (90.7%, *n* = 68) stated they would recommend home BP monitoring to their pregnant patients (Fig. 3). With appropriate measures put into place, they perceived it would be feasible and is the way forward for improving the diagnosis and management of preeclampsia in Ghana.

*“So I think this should be the way to go…Helping [patients] get a monitor, teaching them how to check it properly, and then telling them or educating them on what to do when they get abnormal figures. This will help.” ID 12, Male Junior Resident*

Surveys demonstrated that 81.3% (*n* = 61) of participants would use home BP data to inform their clinical decisions and 89% (*n* = 67) would take immediate action based on elevated home BP values. In interviews, doctors explained they would use home BP values to adjust doses of antihypertensive medications, to differentiate between true hypertension and white coat hypertension, and most importantly, to recommend immediate in-person assessment if a patient measured new or worsening BP elevations at home.

*“If the BP is high, it is high. So if they come [in] or they call that they are checking their BPs and it is high, then you have to act on it… It will affect the clinical management” ID 13, Male Junior Resident*

## Discussion

### Principal findings

Obstetric doctors at a tertiary hospital in urban Ghana hold very positive attitudes towards home BP monitoring in pregnancy. Doctors believe that the many benefits of home BP monitoring far outweigh the challenges to implementation. The primary benefits are improved clinical outcomes, greater patient empowerment and involvement in their care, and more effective healthcare resource utilization. The main barriers are the cost of BP monitors, lack of an efficient patient-provider communication system, and patient health literacy. Doctors emphasized that if the cost of the BP monitor could be subsidized, then the majority of the other barriers could be addressed with patient education and counseling.

### Results in the context of what is known

Few studies have evaluated the utility of home BP monitoring, particularly in pregnancy and in LMICs. Furthermore, there is limited research globally focused on physician attitudes towards the implementation and clinical use of home BP monitoring. Similar to the findings from this qualitative study, studies evaluating home BP monitoring in non-pregnant patients in HIC settings also found patient education and counseling to be a key issue. Despite this barrier, patients and providers had positive attitudes towards home BP monitoring with benefits including convenience, patient empowerment, and trust in BP values [14, 18]. A pilot study in the United Kingdom about home BP monitoring in pregnancy found patient-reported benefits that were similar to those reported by our participants, including patient empowerment and increased engagement in their own healthcare, and that patient willingness and ability to adhere to home monitoring was high [15]. Consistent with clinical benefits reported by participants in our study, a systematic review and meta-analysis of home BP monitoring in pregnancy found a 70% reduction in antepartum hospital admissions and a 50% lower rate of development of preeclampsia associated with use of home BP monitoring [16]. However, recent large-scale RCTs among pregnant women in the United Kingdom did not demonstrate earlier clinical diagnosis of elevated BPs associated with home BP monitoring [20, 21].

### Clinical and policy implications

By describing the current challenges to routine BP monitoring in Ghana, this study emphasizes the need for home BP monitoring in low-resource settings. Home BP monitoring can augment current clinical practice by identifying women with new or worsening elevations of BP at home. Recognizing that false positive BP revelations are possible at home, elevated home values should be followed by subsequent clinical assessment by a healthcare provider. Together, this approach will facilitate earlier diagnosis of HDPs and potential for earlier medical intervention at a healthcare facility. Like other aspects of antenatal care, home BP monitoring should be utilized at the discretion and supervision of healthcare providers and health systems. There is value in wide-scale adoption of home BP monitoring for every pregnant patient, because even low risk pregnancies can develop hypertensive disorders. In addition, there is value in early adoption (in 1st trimester) of home BP monitoring to establish baseline blood pressure values, identify chronic hypertension, distinguish between true and white coat hypertension, and establish patient routines. However, given varying availability of resources and levels of feasibility, protocols in different clinical settings should delineate which populations (all pregnant women or only those with risk factors for development of HDP) may benefit the most from home blood pressure monitoring and at which time point in pregnancy (first trimester or later second trimester) it should be initiated.

We also highlight potential barriers to home BP monitoring in LMIC settings, including lower patient health literacy and numeracy. However, we conclude that with appropriate education and training, all patients could successfully partake in home BP monitoring. Home monitoring programs targeting illiterate patients should consider modifications such as assistance from relatives, use of photographs for training, and use of color-coded results rather than objective BP values. When considering wide-scale implementation of home BP monitoring, doctors identified potential future challenges, including increased patient loads in the hospitals due to greater detection of BP elevations. Thus, implementation approaches should anticipate these potential issues and include appropriate evaluation metrics. In particular, the cost of home BP monitors is an important and addressable barrier. Doctors believe that providing home BP monitors to patients would be more economical than in-patient BP monitoring and suggested using that rationale to advocate for the coverage of home BP monitors by Ghana’s National Health Insurance.

### Research implications

Amidst conflicting evidence regarding the efficacy of home BP monitoring during pregnancy in HIC settings [16, 20, 21], our study emphasizes the differences in the current state of BP monitoring between HICs, which have high compliance with weekly visits in late third trimester, and LMIC settings like Ghana, which have significant barriers to frequent in-person care and monitoring. Participants in our study anticipate that home BP monitoring would result in increased quantity and quality of BP values among high-risk pregnant women in the Ghanaian context. We also demonstrate that the benefits of home BP monitoring extend beyond the potential for earlier diagnosis of elevated BPs, to other important benefits including patient empowerment and improved utilization of limited healthcare resources (i.e. reduced admissions simply for BP monitoring). Together, these findings highlight the significant potential impacts of home BP monitoring in low-resource areas and the critical need for additional studies to be conducted in these highest-risk settings.

### Strengths and limitations

With a diverse sample of different clinical roles and levels of experience, this mixed methods study provides key qualitative and quantitative data on an emerging, clinically important, and understudied topic. Limitations do exist that may impact the interpretation and generalizability of the results. First, a single study site was selected to facilitate a qualitative design that was able to explore thorough and nuanced perspectives. Second, interviews were performed within the hospital compound and the research team included the participants’ peer obstetrics colleague. Thus, participants could have been hesitant to share challenges about the hospital setting or their own clinical practice. However, involvement of a local obstetric doctor in the research team was critical to developing a comprehensive and culturally competent interview guide as well as a tailored sampling strategy. Moreover, interviews were intentionally performed by an American research team member to limit this concern. Surveys were conducted on an anonymous electronic platform, and survey data showed strong agreement with qualitative results. Third, midwives were not included as participants. Given their critical role in obstetric care in Ghana and most LMICs, future research should address midwives’ perspectives.

## Conclusion

Overall, obstetric doctors in Ghana strongly support the implementation of home BP monitoring in Ghana and believe it would be successful and impactful. Obstetric doctors would use patient-measured BP values to make meaningful changes in clinical management and believe home BP monitoring would improve patient outcomes and healthcare system efficiency.

## Supplementary Information

**Additional file: 1.** 

**Additional file: 2.**

## Data Availability

The datasets generated and/or analysed during the current study are not publicly available due to the data (qualitative interview transcripts) containing specific, personal, and detailed information about the participants, but are available from the corresponding author on reasonable request. The important and representative information are available in the quotations and tables available in the manuscript.

## Abbreviations

HDP: Hypertensive disorders of pregnancy
LMICs: low- and middle-income countries
BP: Blood pressure
HICs: High-income countries
KBTH: Korle Bu Teaching Hospital
OBGYN: Obstetrics and Gynaecology
