# Supplementary material for: Benefits and barriers of home blood pressure monitoring in pregnancy: perspectives of obstetric doctors from a Ghanaian tertiary hospital
Source: BMC Pregnancy Childbirth. 2023 Jan 19;23:42. doi: 10.1186/s12884-023-05363-5 (PMC9854160; doi:10.1186/s12884-023-05363-5)
Supplement: Supplementary file 1 — Additional file: 1. [file 12884_2023_5363_MOESM1_ESM.pdf]

Provider Perspectives on Challenges Managing Preeclampsia and the Utility of Home Blood Pressure Monitoring

Dear House Officers, Residents in OBGYN, and Consultants in OBGYN,

You are invited to participate in our study about the provider perspectives on (1) challenges managing preeclampsia and (2) utility of patient-performed home blood pressure monitoring.

Participants are house officers, OBGYN residents, and OBGYN consultants working in the Korle Bu Teaching Hospital. This survey will ask about the following: your experiences with managing preeclampsia, your perspective on your patients performing blood pressure monitoring themselves at home, and how feasible home blood pressure monitoring would be for your patients.

This study is approved by KBTH IRB (KBTH-STC 00098/2021). Benefits of this study include understanding the individual and systemic factors that lead to poor health outcomes due to preeclampsia/ hypertensive disorders of pregnancy in Ghana as well as assessing the utility of home blood pressure monitoring from the provider perspective. There is no more than minimal risk. All responses are anonymous. We will use a study ID number, and will not use your name. Participation is voluntary. The survey will take no longer than 10-15 minutes.

We appreciate your time and perspective!

I agree to participate in the research study. I understand the purpose and nature of this study and I am participating voluntarily. I understand that I can withdraw from the study at any time, without any penalty or consequences.	◯ Yes<br>◯ No

Please select your level of agreement with the following statements about preeclampsia in Ghana.

	Strongly Disagree	Disagree	Neither agree nor disagree	Agree	Strongly Agree
Addressing preeclampsia should be a priority to reduce maternal mortality/ morbidity in Ghana.	◯	◯	◯	◯	◯
Maternal mortality/ morbidity from preeclampsia is preventable.	◯	◯	◯	◯	◯
Delayed diagnosis of preeclampsia is common amongst my patients.	◯	◯	◯	◯	◯
Delayed diagnosis of preeclampsia leads to poor health outcomes amongst my patients.	◯	◯	◯	◯	◯
Early detection of preeclampsia would reduce poor health outcomes amongst my patients.	◯	◯	◯	◯	◯

In some settings, pregnant women check their own blood pressures at home and report abnormal values to their healthcare providers.

Please select your level of agreement with the following statements about home blood pressure monitoring.

	Strongly Disagree	Disagree	Neither agree or disagree	Agree	Strongly Agree
I am aware that high-risk pregnant patients in many countries monitor their blood pressures at home.	○	○	○	○	○
Currently, my pregnant patients monitor their blood pressure at home.	○	○	○	○	○
Home blood pressure monitoring would be feasible in Ghana.	○	○	○	○	○
Home blood pressure monitoring could facilitate early detection and diagnosis of preeclampsia.	○	○	○	○	○
Home blood pressure monitoring could reduce poor health outcomes due to preeclampsia.	○	○	○	○	○
I would recommend home blood pressure monitoring to my patients.	○	○	○	○	○

Please select your level of agreement with the following statements about your patients' attitudes towards preeclampsia and home blood pressure monitoring.

	Strongly Disagree	Disagree	Neither agree or disagree	Agree	Strongly Agree
My patients know about the risks and complications of preeclampsia.	○	○	○	○	○
My patients are interested in taking actions to reduce the risk of developing preeclampsia.	○	○	○	○	○
My patients would be interested in monitoring their blood pressures at home.	○	○	○	○	○
My patients are capable of accurately monitoring their blood pressures at home.	○	○	○	○	○
My patients would follow the recommended schedule to monitor their blood pressures at home.	○	○	○	○	○

Please select your level of agreement with the following statements about your interpretation of patients' home blood pressure measurements.

	Strongly Disagree	Disagree	Neither agree or disagree	Agree	Strongly Agree
I would trust the blood pressures that my patients monitor at home to be accurate.	○	○	○	○	○
I would use my patients' home blood pressures to guide my clinical decision-making.	○	○	○	○	○
I would take immediate action if my patients' home blood pressures were greatly elevated.	○	○	○	○	○

The next set of options describe possible barriers to implementing home blood pressure monitoring in Ghana. Please rate each barrier from 1 (anticipated minimal barrier) to 5 (anticipated maximal barrier).

	1: Minimal Barrier	2	3	4	5: Maximal Barrier
Patient interest in monitoring blood pressures at home	◯	◯	◯	◯	◯
Patient ability to use blood pressure monitor correctly to obtain accurate values	◯	◯	◯	◯	◯
Patient adherence to recommended schedule of blood pressure monitoring	◯	◯	◯	◯	◯
Patient health literacy to recognize elevated blood pressure values	◯	◯	◯	◯	◯
Cost of blood pressure monitors	◯	◯	◯	◯	◯
System for patient-provider communication about abnormal blood pressure values	◯	◯	◯	◯	◯

Please list any other possible barriers you anticipate

____

The next set of questions will ask about your basic demographic information.

What is your clinical role?	◯ House Officer<br>◯ Junior Resident in Obstetrics/Gynaecology<br>◯ Senior Resident in Obstetrics/Gynaecology<br>◯ Consultant in Obstetrics/Gynaecology
Which team are you on?	◯ Team A<br>◯ Team B<br>◯ Team C<br>◯ Team D<br>◯ Team E
What gender do you identify with?	◯ Male<br>◯ Female<br>◯ Other/ Prefer Not To Respond
What is your age?	◯ 20-29<br>◯ 30-39<br>◯ 40-49<br>◯ > 50
How many years have you been practicing as a doctor?	◯ < 1 year<br>◯ 1-5 years<br>◯ 6-10 years<br>◯ 11-20<br>◯ >20 years
On average, how many patients with preeclampsia do you manage weekly?	◯ 0-5<br>◯ 6-10<br>◯ 11-15<br>◯ 16-20<br>◯ >20
On average, how many patients with eclampsia do you manage monthly?	◯ 0<br>◯ 1<br>◯ 2<br>◯ 3<br>◯ 4<br>◯ 5 or higher
