# Supplementary material for: Benefits and barriers of home blood pressure monitoring in pregnancy: perspectives of obstetric doctors from a Ghanaian tertiary hospital
Source: BMC Pregnancy Childbirth. 2023 Jan 19;23:42. doi: 10.1186/s12884-023-05363-5 (PMC9854160; doi:10.1186/s12884-023-05363-5)
Supplement: Supplementary file 2 — Additional file: 2. [file 12884_2023_5363_MOESM2_ESM.pdf]

Interview Guide:

Provider Perspectives on the Utility of Home Blood Pressure Monitoring

Thank you for agreeing to participate in our study about provider perspectives on challenges managing preeclampsia and the utility of home blood pressure monitoring.

There are a few things I will go over with you before starting the interview. First and foremost, this interview is completely confidential. This means I cannot tell anyone about your answers. In addition, your name will not be associated with your responses. Instead, we will assign you a study number so that only those of us running the study know that these are your responses. Please feel free to be honest and open about your experience, as there will be no consequences. If there are any questions that make you uncomfortable or you wish to stop the interview, you can let me know at any point. And lastly, feel free to ask me any clarifying questions if necessary.

There are two components to our study. There is a survey component, which you may have heard about and received a Whatsapp link to. If you have not done so already, we would really appreciate it if you could also complete the survey at your earliest convenience. Right now, we will be doing the interview component, in which I will ask you open-ended interview questions about your experiences managing preeclampsia and thoughts about home blood pressure monitoring. Interviews are expected to last approximately 30-60 minutes.

The interview portion will be audio-recorded for later transcription and analysis. The recording will be stored securely. After they are transcribed, the recordings will be deleted. The recordings and transcriptions will not be linked to your name or any other identifying information.

Before signing the consent form, do you have any questions?

[sign consent form]

I will turn on the recorder now so I can record our discussion. For these questions, the answers can be as long as needed for you to answer them fully.

All participants were asked all bolded questions.

Section 1: Participant Attitudes Towards Home Blood Pressure Monitoring

I would like to ask about your thoughts and perspectives on patients monitoring their own blood pressures (BPs) at home. In some places, pregnant patients often are taught to check their BPs at home, and then they call the clinic if they have elevated BPs or symptoms.

1. **Tell me about your experience with monitoring your patients' BP's during antenatal care?**
    a. **What are the challenges or issues you face?**

2. **Tell me about your experience with your pregnant patients monitoring their own BPs at home.**

   a. **Do any of your patients currently monitor blood pressures at home?**
   b. **Do you recommend home blood pressure monitoring to your patients?**
      i. Why/why not?

3. **Do you think pregnant women monitoring their own BPs at home is feasible in Ghana?**
   a. **Why or why not?**
   b. **Systemic barriers?**
      i. Potential factors to consider include high patient to provider ratio, inadequate patient counseling, no phone triage system, etc.
   c. **Individual patient barriers?**
      i. Potential factors to consider include patient interest, agency, capability, health literacy, cost of blood pressure monitors (if not compensated for), etc.

4. **Do you think patient's monitoring their own BPs at home would be accurate?**
   a. **Why/ why not?**
   b. What factors might make you trust the measurements more?
   c. What factors might make you trust the measurements less?

5. **Would BP values from home blood pressure monitoring affect your clinical management of your patients? In what way?**
   a. **If your patient reports elevated blood pressure measurements at home, what would you do next?**
   b. What factors would motivate you to act immediately?
   c. What factors would prevent you from acting immediately?

6. **If all high risk pregnant women checked their blood pressures at home, what new changes do you expect?**
      i. In your practice?
      ii. In the system?

7. **Do you think home blood pressure monitoring could aid in early detection of preeclampsia? Why/ Why not?**
   a. Could it reduce poor health outcomes? How so?

<u>Section 2: Perceived Patient Attitudes Towards Home Blood Pressure Monitoring</u>

Now, I would now like to ask about how you think your pregnant patients may respond to home blood pressure monitoring.

1. **Are pregnant patients in Ghana involved in their own antenatal care?**
    a. Do pregnant patients understand their antenatal care?
    b. Do pregnant patients want to be involved in their care?

2. **How do you think patients would respond if asked to regularly monitor their own blood pressures at home, given a BP monitor and proper training?**
    a. Do you think asking patients to monitor BPs at home is worthwhile or would it just be an added burden?

3. **What are the barriers to patients adhering to regular blood pressure monitoring? What are the motivators?**
    a. Potential factors to consider include patient interest, agency, capability, health literacy, cost of blood pressure monitors (if not compensated for), etc.

Is there anything else you would like to share about your perspectives on home BP monitoring?

<u>Section 3: Demographics</u>

Clinical Role:
House Officer
Junior Resident in Obstetrics/Gynaecology
Senior Resident in Obstetrics/Gynaecology
Consultant in Obstetrics/Gynaecology

Gender:
Male
Female
Other/ Prefer Not To Respond

Years in practice as a doctor:
<1 year
1-5 years
6-10 years
11-20
>20 years

Average Number of Patients with Preeclampsia Managed Weekly:
0-5
6-10
11-15
16-20
>20

Average Number of Patients with Eclampsia Managed Monthly:
0
1
2
3
4
5

Closing:

"*We have now completed the interview. Thank you so much for your time and for participating. Is there anything you have questions or comments on? Would there be any other provider (house officer, resident, or consultant) that you would recommend we approach to participate in the interview?"*
